# Annotations

**Published:** 1892-01-09

**Authors:** 


### Annotations.

**A Call for Crusaders.**

What is to be done about the African slave trade? Mr. Consul H. H. Johnston has published a brief account of its present condition in the *Graphic*, and has done with his pencil what his pen could not accomplish. He has shown us some of the things which actually take place. For cold-blooded cruelty and wickedness it is probable that the world has never seen anything worse than the things that are now being done in Central Africa every day. If any European were to treat animals with a hundredth part of the incredible cruelty which the Arab slave hunter inflicts upon the negro slave, he would be torn in pieces by the rage of his own neighbours. The writer knows Mr. Johnston's stories to be relations of bare facts. He has near relatives who have lived for many years on the borders of Central Africa, and who have told similar stories to those of Mr. Johnston times without number. The Arab slave hunter professes to be a religious man—he is a Mohammedan. This is what he does in the name of his religion: He goes with strong and well-armed companies to an African village and attacks it; if resistance is offered, says Mr. Johnston, it is almost always in vain. The villagers know, indeed, that resistance is punished by instant death. In a few minutes all who have resisted are killed; all who are still alive are chained; any who have attempted to escape into the bush are brought in dead or alive. If any Mohammedans have been killed in the conflict, the most ruthless vengeance is taken upon the defenceless villagers. For example, the men are tied to trees or stakes, and their limbs lopped off one by one, and finally they are beheaded; the women are killed with the sword; the helpless children are taken up by the feet, swung round and round by their fiendish captors, and then their brains are dashed out against the stone seats of the village squares. When a large company of captives have been gathered together, they are carefully inspected by the Arabs. All those who are aged, deformed, or weakly are separated from the others and made to stand on one side. Their chains are then taken off, and they are told that they may go. They slink off to the bush, but before they get out of sight they are ridden down with shouts of devilish laughter, and shot one by one; or some of them are seized, thrown to the ground, their feet tied together, and then attached by a rope to the saddle or wrist of their captors, who gallop round and round, dragging the living body at the horse's heels until it is nothing but a "shapeless mass of blood and bones." On the march to the slave market, many fall down in intolerable thirst, and are either left to die or are shot, because "dead men tell no tales." Such are some of the horrors of the slave trade in Central Africa. It often strikes the writer as astonishing that no rich man collects an armed force of knightly comrades for a crusade against those iniquitous monsters upon whom the very earth might well cry out for vengeance. It is not a thousand years since the crusaders of England and Europe set out for Palestine to free the Holy Sepulchre from the hands of the unbelievers. The freeing of the Holy Sepulchre was mere Quixotism compared with the noble and civilizing work of exterminating every Arab slave trader from African soil. Not a single man should be left alive. Everyone of them has committed murders without number. The human instinct, which is the parent of all law, insists that such fiends in human shape can have no fitting punishment but that of death. If hundreds of our wealthier young men would band themselves together, and paying their own expenses, put themselves under Consul Johnston's leadership, they would strike a noble blow for civilization, and for the deliverance of captive men and helpless women and children from ills a thousand times worse than death. This may probably be considered advice more suitable for the ninth century than for the nineteenth. But it would not be difficult to show that some things that were done in the ninth century were altogether greater and nobler than some that are done in the nineteenth.

**A Peerless Peer.**

Every scientific man in the world will feel himself honoured in the elevation of Sir William Thomson to the English House of Lords. Sir William has been famous almost ever since he was Second Wrangler and first Smith's Prizeman in 1845. It is one of the curious circumstances in the history of senior wranglers that Sir William should have been second to a man who has never been known to fame. A college companion of the new peer informed the present writer a good many years ago that everybody was astonished when William Thomson only came out second wrangler, and perhaps nobody was more astonished than the senior wrangler himself. Sir William Thomson has had a wonderful, almost a romantic career. He was born at Belfast in 1824, and he is, therefore, not yet at all an old man, as old men go. We hope he may live a score of years or more in which to enjoy his well-earned honours. Sir William, though an Irishman by birth, is not an Irishman by blood, but a Scot, or at least half a Scot. By education and training he is both a Scot and an Englishman. His father was professor of mathematics in the University of Glasgow, and there, at the infantile age of eleven years, the future peer donned the trencher and the red gown of the Scottish Western University. He completed a college course at Glasgow, and then went to Peterhouse, Cambridge, where, as we have said, he graduated second wrangler in 1845, and was immediately afterwards elected to a fellowship. Two years later, that is, at the age of twenty-three, he was made professor of natural philosophy in his father's university of Glasgow, and there he still remains conferring world-wide renown upon his earliest alma mater. To give merely a list of Sir William Thomson's contributions to science would be to fill more space than we have at disposal. But he will always be known for his successful engineering work in connection with the laying of the Atlantic cable in 1866. On that occasion he received the somewhat meagre honour of a knighthood, and at the same time was presented with the freedom of the City of Glasgow. The Royal Society of London has conferred upon him its Royal Medal, and the Royal Society of Edinburgh has given him its Keith Prize. He is honorary LL.D. of the Universities of Cambridge, Edinburgh, and Dublin; and honorary D.C.L. of Oxford. In fact, there is hardly a scientific distinction in the world which has not been conferred upon him; and certainly not one in Great Britain. Now the State has crowned all his other honours by elevating him to the House of Lords, and every thoughtful man will congratulate the House of Lords quite as warmly as he congratulates Sir William Thomson. A new departure has been made by Lord Salisbury, and it is a departure of the most sensible kind. It has long been a ridiculous thing that a born noodle should in many cases sit in the House of Peers whilst a distinguished genius should be compelled to be content with the honours that befit a provincial mayor or a successful soap-maker. Lord Salisbury is a scientific man himself, and so is Mr. Balfour. They know the difference between a man like Sir William Thomson and an hereditary Duke of Dunderhead. We may take it for granted that once the ice has been broken we shall see more of the highest honours the State has to bestow conferred upon men of science. It has long been the desire of many that some of our distinguished physicians should be raised to the House of Lords. Such an act on the part of the Prime Minister would not only gratify a highly meritorious and useful profession, but would be felt to be an act of justice and wisdom by all who take a true measure of the value of different kinds of scientific and public work. It is clear that we are progressing as a State. Mental capacity and achievement are becoming daily more and more valued, and this is a sure indication of the mental capacity and achievement of the valuers. We desire, among thousands of others, to offer our sincere congratulations to the new peer and to the university of which he is so distinguished a professor.

